# Antioxidant and cytokine modulation in PCOS rats protected with probiotics, myo-inositol, and herbal extracts

**DOI:** 10.17221/116/2024-VETMED

**Published:** 2025-06-27

**Authors:** Abdelkader Ahmed Zaki, Naif Muqbil Alharbi, Tariq Ibrahim Almundarij, Saleh Mohammed Albarrak

**Affiliations:** ^1^Department of Biomedical Sciences, College of Veterinary Medicine, Qassim University, Buraydah, Saudi Arabia; ^2^Department of Physiology, Faculty of Veterinary Medicine, Cairo University, Giza, Egypt; ^3^National Center for the Prevention & Control of Plants Pests & Animal Diseases, Buraydah, Qassim Region, Saudi Arabia; ^4^Department of Pathology and Laboratory Diagnosis, Collage of Veterinary Medicine, Qassim University, Buraydah, Saudi Arabia

**Keywords:** antioxidants, ELISA, immunity, myo-inositol, polycystic ovarian syndrome

## Abstract

This study investigated the pathophysiology of polycystic ovarian syndrome (PCOS) and evaluated the protective effects of various treatments in immature female Wistar rats (*N* = 48). The rats were divided into 6 groups: Olive oil injection (negative control, G1); testosterone propionate (TP)-induced PCOS (G2); probiotic + TP (G3); myo-inositol (myo-ins) + TP (G4); *U. dioica* extract + TP (G5); *W. somnifera* extract + TP (G6). The body weight, body weight gain, and percentage gain were measured weekly and then transformed using the base-10 logarithm (log_10_). TNF-α, IL-4, IL-10, and IL-17 were weekly measured using ELISA kits. Glutathione peroxidase (GSH-Px), superoxide dismutase (SOD), and catalase (CAT) were analysed in the serum and liver extracts. The *W. somnifera* significantly reduced the TNF-α levels (*P* < 0.01). The probiotic and myo-ins significantly elevated the IL-10 levels (*P* < 0.01). Both plant extracts moderately restored the IL-10 levels. The probiotic and *U. dioica* administration significantly reduced the IL-17 levels (*P* < 0.01). The *W. somnifera* administration also decreased the IL-17 levels, though the effect was less pronounced than that of *U. dioica*. The probiotic, myo-ins, and *W. somnifera* groups exhibited enhanced CAT activity (*P* < 0.05). *W. somnifera* showed significant increases in the SOD and GSH-Px activities (*P* < 0.01), showing the most dramatic improvement. The use of these four treatments as a monotherapy in this study resulted in different changes. Therefore, further investigation is necessary to evaluate the protective effects of combining duos or trios of these treatments against this disease.

Multiple variables have been discovered in the pathophysiology of polycystic ovarian syndrome (PCOS), making it a complex condition. This syndrome is caused by a combination of hereditary factors (Liu et al. 2021), immune system abnormalities (Rudnicka et al. 2021), and environmental factors (Teede et al. 2018). These are linked to an increase in several inflammatory agents and mediators, which are another component contributing to the pathophysiology of this disease. However, the current understanding has a strong bias toward oxidative stress (OS), low-grade inflammation, and their interdependence, as the key drivers of the signalling needed to develop PCOS (Ajibare et al. 2023). Hyperandrogenism, persistent anovulation, insulin resistance, and polycystic ovary morphology are the hallmarks of the condition, which also affects the endocrine, metabolic, and reproductive systems (Xu and Qiao 2022).

The PCOS rat’s unbalanced hormone concentrations, lipid profile, and antioxidant status are improved by the antioxidant and anti-inflammatory properties of plant extracts (Adelakun et al. 2024). Herbal compounds have been identified as one of the most essential antioxidants in recent years, and numerous studies have been conducted on their use (Mazloom et al. 2019). Due to the high cost of chemical drugs, drug resistance, and several negative effects, medicinal plants with antioxidant and anti-inflammatory effects can be a great substitute for chemical pharmaceuticals. They can be used as an efficient medication in the management and treatment of PCOS.

Research has demonstrated the anti-inflammatory, antioxidant, and anti-diabetic properties of inositol (Arefhosseini et al. 2023). The most common inositol found in living things is myo-inositol, which can also be considered a lipid. It exists as a sugar alcohol and in a free form (Antonowski et al. 2022). It functions both as a primary and secondary metabolite (Greff et al. 2023). The use of myo-ins in PCOS protection has long been disputed. As a result, the goal of the current study is to assess the safety and effectiveness of myo-ins in protecting against PCOS.

Emerging research has demonstrated a close relationship between metabolic disorders such as PCOS and the gut microbiome. Probiotics can help the gut microbiota become more diverse again, and, in PCOS-like mice, the healing of gut microbiota problems enhances the ability to reproduce. The results from the present investigation will contribute to our understanding of the effects of gut microbiota on the inflammatory and oxidative changes associated with PCOS.

Known by many names, such as common or stinging nettle, *Urtica dioica* (*U. dioica*) is a multipurpose plant that has been utilised for ages as a wild herb. *U. dioica* is said to be an ancient medicinal herb used to treat lumbago and arthritis in people, as well as its ability to improve blood flow and provide warmth to the affected part of the body (Upton 2013). The scientific community has taken notice of nettle due to its anti-diabetic, anti-inflammatory, antiviral, anti-ulcer, immune-stimulating, anti-infectious, and antioxidant characteristics (Bhusal et al. 2022). The primary constituents of the nettle plant, flavonoids, phenolic acids, lignans, and phytosterols, are responsible for its biological activities (Taheri et al. 2022).

Ashwagandha*, Withania somnifera* (*W. somnifera*), is a traditional medicinal herb used to treat a wide range of ailments. Ashwagandha, commonly called Indian ginseng, is a member of the *Solanaceae* family, which also includes the *W. somnifera* plant. Ashwagandha is the “Queen of herbs” due to its numerous beneficial effects. *In vitro* and *in vivo* experiments were carried out to evaluate the efficacy of the root extract. This herb offers relief from a variety of conditions and illnesses, including diabetes, fatigue, seizures, and many others (Dutta et al. 2019). The administration of an ashwagandha extract, which is thought to be a potent plant with health-promoting qualities, had a mitigating impact on testicular toxicity (Awad 2022).

The current investigation aims to examine whether inflammatory cytokines and oxidative stress biomarkers contribute to the pathogenesis of infertility in the PCOS rat model. Different lines of protection were applied to rats to obtain a possible solution for PCOS.

## MATERIAL AND METHODS

### Ethics

The current work was permitted by the Animal Ethics Committee of Qassim University (No. 24-12-02). All experimental procedures were conducted in accordance with institutional guidelines for animal welfare and care.

### Experimental design

Forty-eight immature female (30 ± 10 g body weight) Wistar rats were transported to the experimental animal unit under hygienic rearing conditions. The animals were kept in controlled conditions of temperature, humidity, and light/dark cycles (25 °C, 55%, and 12 : 12 h, respectively). The commercial diet was obtained from the General Company of Feed Silo and Powder Mint. The diet was formulated to furnish the requirements and was delivered with water *ad libitum*. The feed ingredients included soybean (18.0%), ground yellow corn (21.5%), barley (10.0%), wheat bran (14.0%), hay (29.5%), protein supplement (5.0%) and vitamins and a minerals mixture were formulated to furnish all the nutritional requirements of the rats.

### Preparation of the *U. dioica* and *W. somnifera* extracts

The nettle’s aerial portions were harvested, washed, and dried at 55 °C using an air oven. After grinding, the harvested powder was then combined with ethanol in a 1 : 10 ratio in the dark for extraction. Following extraction, the ethanol was removed from the mixture using a rotary evaporator, and the extract was stored at –20 °C till use (Bandariyan et al. 2021). The Ashwagandha aerial portions were harvested. An electronic grinder was used to create a coarse powder; 100 g of this powder was utilised for extraction in 50% distilled water. The liquid extract was chilled, filtered through Whatman filter paper, and then allowed to evaporate over a water bath (60 °C) until dry (Saiyed et al. 2016). The extract was stored at –20 °C till use.

### Treatment protocol

Immature rats at the age of 21 days were divided into 6 groups: The first group was injected slowly intraperitoneally with 0.5 ml olive oil in water (1 : 1) for 35 days and considered the negative control group (G1). The second group (G2) was injected intraperitoneally for 35 days with 10 mg/kg body weight of testosterone propionate (TP-VIROME; Sven Pharma, Leverkusen, Germany) (Chaudhari and Nampoothiri 2017). The third group (G3) was injected intraperitoneally for 35 days with 10 mg/kg body weight of TP + probiotic 2 ml/day orally (2.0 × 10^8^ colony-forming units (CFU)/ml containing a mixture of *Bifidobacterium*, *Enterococcus faecalis*, *Lactobacillus acidophilus*, *Bifidobacterium lactis*, *Bifidobacterium longum*, *Lactobacillus rhamnosus*, *Bifidobacterium breve*,* Lactobacillus casei*, *Lactobacillus salivarius*, and *Lactobacillus plantarum* for 35 days (Gulan et al. 2019). The fourth group (G4) received TP + myo-ins 420 mg/kg in 2 ml of H_2_O orally for 21 days. The fifth group (G5) received TP + *U. dioica* extracts 200 mg/kg orally for 28 days (Taheri et al. 2022). The last group (G6) received TP + *W. somnifera* extracts of 200 mg/kg orally for 30 days (Bhargavan et al. 2015).

### Measurement of the body weight

The body weight (BW) and body weight gain percent (BWG%) were recorded weekly for each group throughout the experimental period and then transformed using the base-10 logarithm (log_10_). At the end of the experiment, a microscopic examination of the major cell types in the vaginal smears was conducted to determine the stage of cyclicity.

### Blood and tissue collection

**Figure 1:**
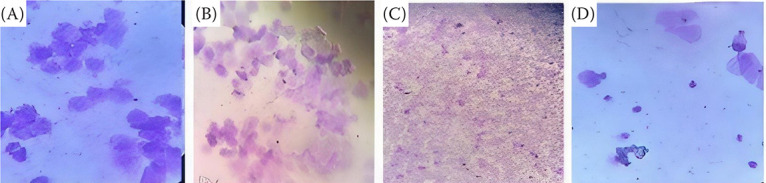
Phases of the oestrous cycle by the vaginal smear (A) Proestrus phase, epithelial cells; (B) Oestrus phase, cornified cells; (C) Met oestrus phase, leucocytes; (D) Dioestrus phase, few leucocytes and cornified cells

Blood samples were collected via the inner canthus of the eye using capillary tubes every week for 30 days. Part of the blood samples was used for the extraction of serum by centrifugation at 605 *g* for 15 minutes. The collected sera were labelled and deeply frozen for the pending analysis. On the last day, all the rats were fasted overnight, anaesthetised, bled from the inner canthus, and sacrificed. The spleen, liver, and ovaries were excised for a histological analysis (Figure 1).

### Cytokines quantification

The cytokine quantification was conducted with quantitative enzyme-linked immunosorbent assay (ELISA) kits purchased from Sunglung Biotech Co., Ltd. (No.35; Tainan, Taiwan). The levels of TNF-α, IL-4, IL-10 and IL-17 in the sera were quantified in accordance with the manufacturer’s instructions (Cat. Nos. SL0722Ra, EL0036Ra, EL0032Ra, EL0026Ra, respectively). The samples were examined in triplicate. For every plate, a standard curve was created to illustrate the relationship between the standards’ concentrations and absorbance values. The cytokine concentrations for each sample were presented as ng/l or pg/ml.

### Determination of the antioxidant activity of the liver tissue

The liver specimens were dissected from each rat, washed with cold saline, and homogenised with ice-cold Tris-HCl (0.1 M, pH 7.4) in a ratio of 1 : 10 (w/v) (Erejuwa et al. 2010). The homogenates were stored at –20 °C until use. The suspended mixture was centrifuged at 650 *g* for 15 min at 4 °C in a cold centrifuge. The obtained supernatant was used for the assay of GSH-Px, SOD and CAT activities. The measurement of protein content of the supernatants was conducted by total protein kits (LOT TP 1010; Spectrum, Cairo, Egypt). The enzyme activities were expressed as U/mg protein. The glucose determination was performed using the enzymatic colorimetric method according to the standardised method described by Menarini Diagnostics, Lot No. 9506 (Firenze Capitale d'Italia).

### Histological analysis

The histopathological protocol was carried out as recorded by Suvarna et al. (2018). After the post-mortem examination, the tissue samples were collected, with a primary focus on the ovary and spleen. The ovaries were cleaned of any adherent connective fat tissue, followed by fixation of the collected tissues in a 10% formaldehyde buffer for histological examination. The tissues were embedded in paraffin, cut into 8-μm sections, stained with haematoxylin and eosin, and examined later under a light microscope.

### Statistical analysis

Data values were represented as the means with standard errors. The data for the body gain were initially calculated as a percentage and then transformed using the base-10 logarithm (log_10_). This transformation was made to enable the data to be analysed more effectively using the SPSS software v20 (USA). A straightforward one-way analysis of variance (ANOVA) test was conducted with the SAS program v20 (SAS, USA) for each measured parameter. Tukey’s HSD test was used in a post hoc analysis to compare the control negative group to the other experimental groups and the control positive group (model) with the protection groups.

The following model was applied:

$$Y_{ijm}=\;\text{μ}\;+\;T_{i}\;+\;e_{ij}$$ (1)

where:

μ – overall mean;

*T_i_* – random effect of the treatment group;

*e_ij_* – random error.

## RESULTS

### Body weight improvements

Table 1 presents a comparison of the body weight (log-transformed, denoted as logBW) across the different experimental groups over four weeks (W1 to W4). The table also includes the results of Tukey’s HSD test, which is used to determine significant differences between the group means. For each week, the mean logBW values and their standard errors are provided for each group. The “*P *≥” column indicates the *P*-values from Tukey’s HSD test, with “ns” denoting the non-significant results and specific *P*-values indicating significant differences. In Week 1 (W1), there were no significant differences between the groups (*P* ≥ 0.05). From Week 2 (W2) onwards, significant differences were observed (*P* < 0.05). The highest mean logBW values in each week are indicated by the superscript letter “a”, and groups sharing the same letter are not significantly different from each other. Significant differences were observed, with the *U. dioica* and *W. somnifera* groups consistently showing higher body weight compared to the control negative and probiotic groups. The results suggest that the *U. dioica* and *W. somnifera* treatments may have a positive effect on the body weight over time compared to the control and probiotic treatment.

**Table 1:** Comparison of the body weight across the different experimental groups with Tukey’s HSD test results

Trait	Control		Probiotic	Myo-ins	*U. dioica*	*W. somnifera*	*P* ≥
	negative	PCOS model					
logBW (W1)	1.29 ± 0.03	1.20 ± 0.03	1.22 ± 0.01	1.20 ± 0.03	1.24 ± 0.02	1.14 ± 0.05	ns
logBW (W2)	1.50 ± 0.02	1.48^b^ ± 0.03	1.50^b^ ± 0.01	1.56^b^ ± 0.01	1.56^b^ ± 0.04	1.68^a^ ± 0.02	0.000
logBW(W3)	1.81 ± 0.01	1.82^b^ ± 0.02	1.83^b^ ± 0.01	1.92^a^ ± 0.01	1.94^a^ ± 0.01	1.92^a^ ± 0.01	0.000
logBW (W4)	1.97 ± 0.01	1.98^b^ ± 0.01	2.00^b^ ± 0.01	2.07^a^ ± 0.02	2.09^a^ ± 0.02	2.00^b^ ± 0.01	0.000

^a,b^Values with different superscript letters within the same row are significantly different (Tukey’s HSD test, *P* < 0.05); “a” represents the highest mean value in each row; Groups sharing the same letter are not significantly different

BW = body weight; myo-ins = myo-inositol; ns = not significant; PCOS = polycystic ovarian syndrome; *U. dioica* = *Urtica dioica*; W1–4 = week 1 to 4; *W. somnifera* = *Withania somnifera*

### Concentrations of the inflammatory and anti-inflammatory cytokine

Table 2 highlights the impact of various interventions, probiotic, myo-ins, *U. dioica*, and *W. somnifera* on the levels of inflammatory cytokines in a PCOS-induced immature rat model. The PCOS group exhibited a significant elevation in the TNF-α levels (20.894 ± 0.456 ng/l) compared to the control group (9.327 ± 2.639 ng/l). Both the probiotic (8.515 ± 2.751 ng/l) and myo-ins (13.51 ± 3.53 ng/l) treatments showed a (*P* < 0.05) reduction in the TNF-α levels compared to the PCOS group, with myo-ins demonstrating a more pronounced effect. Compared to the PCOS group, the *U. dioica* group exhibited no reduction in the TNF-α (19.160 ± 1.35 ng/l), while *W. somnifera* significantly (*P* < 0.001) reduced the TNF-α levels (4.80 ± 1.620 ng/l).

**Table 2:** Cytokine concentration of the PCOS immature rat model protected with probiotic, myo-ins and extracts of *U. dioica* and *W. somnifera*

Parameters	Groups						
	negative *N* = 8	positive (PCOS) *N* = 8	probiotic *N* = 8	myo-ins *N* = 8	*U. dioica* *N* = 8	*W. somnifera* *N* = 8	*P* ≥
TNF- α (ng/l)	9.327^**^ ± 2.639	20.894^a^ ± 0.456	8.515^b^ ± 2.751	13.51^ab^ ± 3.53	19.160^a^ ± 1.35	4.80^b^ ± 1.62	0.000
IL-4 (pg/ml)	7.707 ± 1.523	5.421 ± 1.696	12.775 ± 2.878	10.020 ± 3.530	15.700 ± 5.040	11.940 ± 2.760	ns
IL-10 (pg/ml)	7.45^*^ ± 1.71	2.536^d^ ± 0.207	15.736^a^ ± 0.759	11.121^b^ ± 0.770	4.896^cd^ ± 0.543	8.34^bc^ ± 1.610	0.000
IL-17 (pg/ml)	2.463^**^ ± 0.252	11.70^a^ ± 2.26	3.035^b^ ± 0.597^c^	4.530^b^ ± 1.530	3.620^b^ ± 1.250	7.396^ab^ ± 0.907	0.000

Values are the means ± SEM

*^,^**Values of the negative group were significantly different compared with the positive control group at *P* < 0.05 and *P* < 0.01, respectively; ^a–d^Values with different superscript letters within the same row are significantly different (Tukey’s HSD test, *P* < 0.05); “a” represents the highest mean value in each row; Groups sharing the same letter are not significantly different

IL-4 = interleukin-4; IL-10 = interleukin-10; IL-17 = interleukin-17; myo-ins = myo-inositol; ns = not significant; PCOS = polycystic ovary syndrome; TNF-α = tumour necrosis factor-alpha; *U. dioica* = *Urtica dioica; W. somnifera* = *Withania somnifera*

In PCOS, the IL-4 levels were not significantly different (5.421 ± 1.696 pg/ml) when compared to the control group (7.707 ± 1.523 pg/ml). The PCOS rat treatment with the probiotic, myo-ins, *U. dioica* or *W. somnifera* extract enhanced the IL-4 levels, though to a non-statistically significant extent (12.775 ± 2.878 pg/ml, 10.020 ± 3.530 pg/ml, 15.700 ± 5.040 pg/ml, and 11.940 ± 2.760 pg/ml, respectively).

The PCOS group showed significantly (*P* < 0.05) lower IL-10 levels (2.536 ± 0.207 pg/ml) compared to the control group (7.45 ± 1.71 pg/ml). The probiotics significantly (*P* < 0.001) elevated the IL-10 levels (15.736 ± 0.759 pg/ml). Myo-ins also had a significant (*P* < 0.001) positive impact on the IL-10 levels (11.121 ± 1.770 pg/ml). Both *U. dioica* and *W. somnifera* moderately restored the IL-10 levels with the *W. somnifera* treatment being statistically different (*P* < 0.01) (4.896 ± 0.543 pg/ml and 8.340 ± 1.610 pg/ml, respectively). The PCOS group showed a significant increase (*P* < 0.01) in the IL-17 levels (11.70 ± 2.26 pg/ml) compared to the control group (2.463 ± 0.252 pg/ml). The probiotic administration significantly (*P* < 0.001) reduced the IL-17 levels (3.035 ± 0.597 pg/ml). Myo-ins (4.530 ± 1.530 pg/ml) had a similar, though less pronounced, effect (*P* < 0.01). The *U. dioica* group showed a substantial (*P* < 0.01) reduction in the IL-17 levels (3.620 ± 1.250 pg/ml). *W. somnifera* also decreased the IL-17 levels (7.396 ± 0.907 pg/ml), though the effect was not statistically different than the PCOS group.

### Antioxidant activities and glucose concentrations of the serum

Table 3 presents the activities of the key antioxidant enzymes and glucose levels in the serum of the PCOS rat model protected administrated with probiotics, myo-ins, *U. dioica*, and *W. somnifera*, in comparison to the to the control and the untreated PCOS groups.

**Table 3:** Antioxidant activities and glucose concentration of the serum of the PCOS immature rat model protected with the probiotic, myo-ins and extracts of *U. dioica* and *W. somnifera*

Parameters	Groups						
	negative *N* = 8	positive (PCOS) *N* = 8	probiotic *N* = 8	myo-ins *N* = 8	*U. dioica* *N* = 8	*W. somnifera* *N* = 8	*P *≥
CAT (U/l)	204.98** ± 25.23	108.5^b^ ± 11.8	265.2^a^ ± 21.500	266.7^a^ ± 89.10	221.8^b^ ± 48.310	269.01^a^ ± 9.345	0.000
GSH-Px (mU/l)	37.428 ± 0.210	38.103^a^ ± 0.132	36.864^c^ ± 0.162	37.759^ab^ ± 0.154	37.441^ab^ ± 0.305	37.091^ab^ ± 0.065	0.000
SOD (U/ml)	131.09* ± 19.69	97.21^c^ ± 8.458	142.0^bc^ ± 15.400^c^	165.54^bc^ ± 2.820	164.66^b^ ± 9.810	251.5^a^ ± 27.000	0.000
Glucose (mmol/l)	5.53* ± 0.02	8.71^a^ ± 1.15	4.91^b^ ± 0.07	4.88^b^ ± 0.34	5.25^b^ ± 0.13	4.21^b^ ± 0.01	0.000

Values are the means ± SEM

*^,^**Values of the negative group were significantly different compared with the positive control group at *P* < 0.05 and *P* < 0.01, respectively; ^a–c^Values with different superscript letters within the same row are significantly different (Tukey’s HSD test, *P* < 0.05); “a” represents the highest mean value in each row; Groups sharing the same letter are not significantly different

CAT = catalase; GSH-Px = glutathione peroxidase; myo-ins = myo-inositol; PCOS = polycystic ovary syndrome; SOD = superoxide dismutase; *U. dioica* = *Urtica dioica; W. somnifera* = *Withania somnifera*

The PCOS group showed a significant (*P* < 0.01) reduction in the CAT activity (108.5 ± 11.8 U/l) compared to the control group (204.98 ± 25.230 U/l). Compared to the untreated PCOS group, the probiotic group exhibited significantly (*P* < 0.05) enhanced CAT activity (265.2 ± 21.500 U/l), surpassing even the control group’s levels. The myo-ins supplementation also significantly (*P* < 0.05) elevated the CAT activity (266.7 ± 89.10 U/l). Both *U. dioica* (221.8 ± 48.310 U/l) and *W. somnifera* (269.01 ± 9.345 U/l) improved the CAT activity, but only *W. somnifera* was of statistical significance (*P* < 0.05). The PCOS group was not different from the control group regarding the GSH-Px activity (38.103 ± 0.132 mU/l and 37.428 ± 0.210 mU/l, respectively).

The supplementation with the probiotic, myo-ins, *U. dioica,* or *W. somnifera* had no impact on the GSH-Px activity (36.864 ± 0.162 mU/l, 37.759 ± 0.154 mU/l, 37.441 ± 0.305 mU/l and 37.091 ± 0.065 mU/l, respectively) compared to the PCOS group. The PCOS group exhibited a substantial (*P* < 0.05) reduction in the SOD activity (97.21 ± 8.458 U/ml) compared to the control group (131.09 ± 19.69 U/ml). The probiotics significantly (*P* < 0.001) restored the SOD activity (142.0 ± 15.400 U/ml). Myo-ins and *U. dioica* also significantly (*P* < 0.05) improved the SOD activity (165.54 ± 2.820 U/ml and 164.66 ± 9.810 U/ml). *W. somnifera* also showed a significant (*P* < 0.001) increase in the SOD activity (251.5 ± 27.000 U/ml), showing the most dramatic improvement.

The PCOS group exhibited significantly (*P *< 0.05) elevated glucose levels ((8.71 ± 1.15 mmol/l) com-pared to the control group (5.53 ± 0.02 mmol/l). The probiotics significantly (*P* < 0.001) reduced the glucose levels (4.91 ± 0.07 mmol/l), bringing them close to normal levels. Myo-ins also significantly (*P* < 0.001) lowered the glucose levels (4.88 ± 0.34 mmol/l). Both *U. dioica* (5.25 ± 0.13 mmol/l) and *W. somnifera* (4.21 ± 0.01 mmol/l) showed significant glucose-lowering effects with *W. somnifera* producing the most dramatic reduction (*P* < 0.01 and *P* < 0.001, respectively).

### Antioxidant activities of liver extracts

Table 4 presents a detailed comparison of the antioxidant enzyme activities in the liver extracts of the immature PCOS model rats protected by various interventions. The control group exhibited a mean catalase activity of 24.817 ± 3.623 U/l, representing the baseline antioxidant defence of healthy liver tissue. The PCOS group shows a lower CAT activity of 11.410 ± 4.336 U/l. The probiotic treatment increased the CAT activity to 18.287 ± 2.436 U/l. Myo-ins significantly (*P* < 0.001) increased the CAT activity to 21.280 ± 2.866 U/l, showing a significant improvement over the PCOS group and approaching the near-normal levels seen in the control group. The CAT activity of the *U. dioica* group did not differ from the PCOS group (14.037 ± 3.471 U/l). *W. somnifera* slightly increased the CAT activity to 16.443 ± 3.724 U/l, which was not significantly different from the PCOS group.

**Table 4:** Antioxidant activities of the liver extract of the PCOS immature rat model protected with probiotic, myo-ins and extracts of *U. dioica* and *W. somnifera*

Parameters	Groups						
	negative *N* = 8	positive (PCOS) *N* = 8	probiotic *N* = 8	myo-ins *N* = 8	*U. dioica* *N* = 8	*W. somnifera* *N* = 8	*P *≥
CAT (U/mg protein)	24.817 ± 3.623	11.410 ± 4.336	18.287 ± 2.436	21.280 ± 2.866	14.037 ± 3.471	16.443 ± 3.724	ns
SOD (U/mg protein)	10.015** ± 2.519	3.72 ± 0.803	5.430 ± 1.027 8	7.872 ± 2.036 1	9.629 ± 2.029 5	4.961 ± 1.046 6	ns
GSH-Px (mU/mg protein)	57.77* ± 5.01	38.17 ± 3.22	45.51 ± 4.10	45.14 ± 5.36	41.87 ± 1.03	52.030 ± 5.959	ns

Values are the means ± SEM

*^,^**Values of the negative group were significantly different compared with the positive control group at *P* < 0.05 and *P* < 0.01, respectively

CAT = catalase; GSH-Px = glutathione peroxidase; myo-ins = myo-inositol; ns = not significant; PCOS = polycystic ovary syndrome; SOD = superoxide dismutase; *U. dioica* = *Urtica dioica; W. somnifera* = *Withania somnifera*

The control group shows a SOD activity of 10.015 ± 2.519 U/ml, providing a reference for the antioxidant status of healthy liver tissue. In the PCOS group, the SOD activity drops dramatically to 3.72 ± 0.803 U/ml (*P* < 0.001). The probiotic treatment increases SOD activity to 5.43 ± 1.027 8 U/ml, representing an improvement over the PCOS group, although it remains significantly lower than the control group’s values. Myo-ins also improves the SOD activity to 7.87 ± 2.036 1 U/ml, which is higher than the PCOS and probiotic groups, but still lower than the control group. *U. dioica* dramatically (*P* < 0.01) boosts the SOD levels to 9.629 ± 2.029 5 U/ml, almost reaching the control levels. *W. somnifera* also shows improvement in the SOD activity (4.96 ± 1.046 6 U/ml), but the increase was smaller compared to the other treatment groups, suggesting moderate antioxidant benefits for this particular enzyme.

The control group demonstrates a GSH-Px activity of 57.77 ± 5.01 mU/l, indicating a healthy baseline for the liver antioxidant defence. In the PCOS group, the GSH-Px levels drop to 38.17 ± 3.22 mU/l (*P* < 0.05). The probiotic treatment increases the GSH-Px activity to 45.51 ± 4.10 mU/l. Myo-ins boosts GSH-Px activity to 45.14 ± 5.36 mU/l, comparable to the probiotics, indicating that both interventions improve the liver’s capacity to deal with OS, although the difference was not statistically significant. *U. dioica* improved the GSH-Px activity to 41.87 ± 1.03 mU/l, which is the closest to the control group levels among all the treatments. *W. somnifera* also showed an increase in the GSH-Px activity to 52.03 ± 5.959 mU/l.

### Ovary and spleen histology

**Figure 2:**
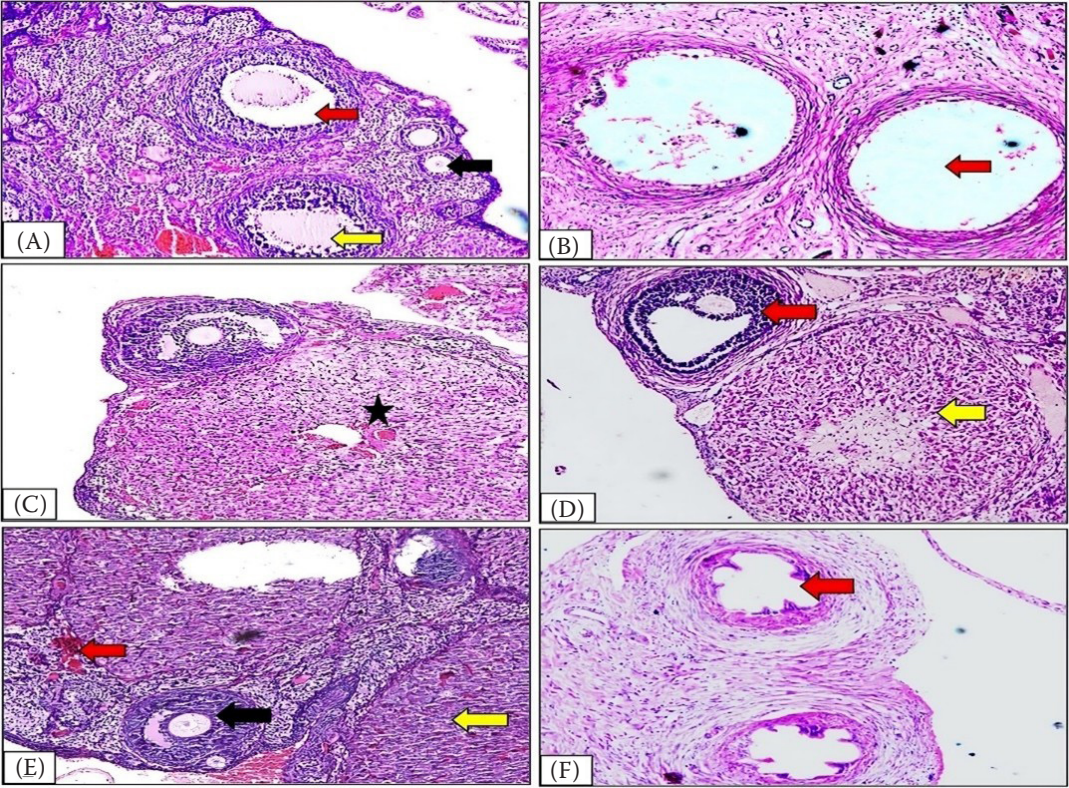
Photomicrograph of ovarian tissues of different experimental groups Photomicrograph from the ovary of immature rats injected with olive oil (A) showed normal, well-developed mature follicles. Ovary showing presence of PF (black arrow), SF (red arrow), and TF (yellow arrow). (B) TP displayed the existence of a follicular cyst (red arrow) associated with a histopathological change. (C) TP + probiotics illustrated normal corpora lutea (star) and the presence of healthy ovarian follicles. (D) TP+ myo-ins illustrated the complete disappearance of the ovarian cysts and normal corpora lutea (yellow arrow) and presence of healthy ovarian follicles in different stages (red arrow). (E) TP + *U. dioica* extracts illustrated the continued existence of medium-sized ovarian follicular cysts (black arrow) and congestion in the ovarian blood vessels (red arrow) with severe multifocal haemorrhages (yellow arrow). (F) TP + *W. somnifera* extracts displayed normally developed follicles of different sizes (red arrow). H&E, ×200 Myo-ins = myo-inositol; PF = primordial follicles; SF = secondary follicles; TF = tertiary growing follicles; TP = testosterone propionate; *U. dioica* = *Urtica dioica*; *W. somnifera* = *Withania somnifera*

The ovaries of the rats in the negative control group show normal, well-developed mature follicles with a balanced distribution of primordial, secondary, and tertiary follicles (Figure 2A). The ovary of a female rat treated with TP showed the presence of a follicular cyst with exfoliation, degeneration, and cell necrosis (Figure 2B). In the probiotic group, the ovary showed the complete disappearance of the follicular cysts that were observed in the previous group treated with TP alone. The presence of normal, well-developed corpora lutea was noted. The presence of healthy ovarian follicles in different stages of development was also observed (Figure 2C). The histological examination of the ovary treated with TP and myo-ins showed the complete disappearance of ovarian cysts, as well as the presence of normal, well-developed corpora lutea and healthy ovarian follicles in different stages of development (Figure 2D). The histological examination of the ovary of a female rat treated with TP and *U. dioica* extracts revealed the presence of a medium-sized follicular cyst, indicating that the treatment did not fully address the condition. There is evidence of congestion in the ovarian blood vessels, suggesting circulatory issues within the ovary. Severe multifocal haemorrhages (bleeding) were also observed in multiple corpora lutea (Figure 2E). The histological examination of the ovary of a female rat treated with TP and *W. somnifera* extracts revealed the complete disappearance of follicular cysts. The ovary exhibits normal, well-developed follicles in various stages of development, indicating healthy follicular maturation (Figure 2F).

**Figure 3:**
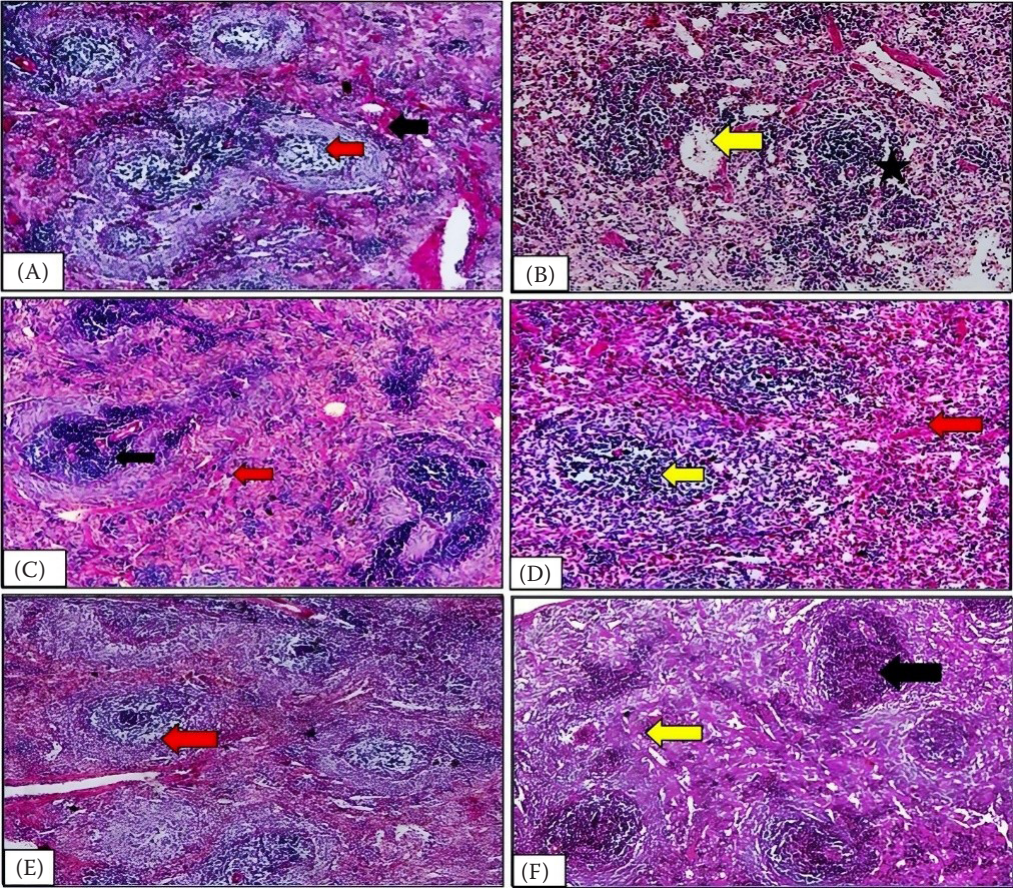
Photomicrograph of spleen of different experimental groups Photomicrograph from the spleen of immature rats of the negative control group injected with olive oil (A) showed normal, well-developed lymphoid follicles in the white pulp (red arrow) with a normal splenic artery (black arrows). (B) Splenic tissue (yellow arrow) exhibits moderate lymphoid depletion in the lymphoid follicles of the white pulp (star) by TP. (C) TP + probiotic showed mild lymphoid depletion in the lymphoid follicles of the white pulp (black arrow) and normal splenic artery (red arrow). (D) TP + myo-ins showed moderate lymphoid depletion in the lymphoid follicles of the white pulp (yellow arrow). (E) TP + *U. dioica* extracts showed splenic tissue with normal impacted lymphoid follicles in the white pulp (red arrow) and a normal splenic artery. (F) TP + *W. somnifera* extracts showed splenic tissue with normal impacted lymphoid follicles in the white pulp (black arrow) and a normal splenic artery (yellow arrow). H&E, ×40 Myo-ins = myo-inositol; TP = testosterone propionate; *U. dioica* = *Urtica dioica*; *W. somnifera* = *Withania somnifera*

The negative control group, treated with olive oil, exhibits a normal splenic architecture with well-developed lymphoid follicles (Figure 3A). The administration of TP appears to have a suppressive effect on the lymphoid tissue within the spleen (Figure 3B). The spleen of a rat treated with TP and a probiotic exhibited lymphoid follicles within the white pulp, accompanied by a mild reduction in lymphoid cells. The splenic artery appears to be structurally normal (Figure 3C). The histology of the spleen of a rat treated with TP and myo-ins exhibited lymphoid follicles within the white pulp of the spleen, showing a moderate reduction in lymphoid cells (Figure 3D). The splenic artery appears to be structurally normal. The combination of TP and myo-ins appears to have a suppressive effect on the lymphoid tissue within the spleen, similar to the group treated with TP alone. The histological image depicts the spleen of a rat treated with TP and *U. dioica* extracts (Figure 3E). The lymphoid follicles within the white pulp of the spleen appear normal and intact, with no significant signs of depletion or atrophy. The combination of TP and *W. somnifera* extracts appears to have a minimal or no effect on the lymphoid tissue within the spleen in this case (Figure 3F). The spleen maintains its normal architecture and the integrity of lymphoid follicles.

## DISCUSSION

PCOS is a prevalent endocrine disorder characterised by a chronic low-grade inflammation. In the present study, the levels of specific cytokines, including TNF-α, IL-4, IL-10, and IL-17, were assessed. Cytokines play a crucial role in immune responses, and their imbalance has been linked to the pathogenesis of PCOS. TNF-α is a pro-inflammatory cytokine closely linked to the PCOS pathology due to its role in promoting inflammation, insulin resistance, and ovarian dysfunction. In this study, the PCOS group exhibited a significant elevation in TNF-α levels compared to the control group, indicating heightened inflammation in PCOS conditions. TNF-α is recognised as a mediator of insulin resistance, which implies that it could be a factor in PCOS metabolic dysfunction (Escobar-Morreale et al. 2011). The PCOS group showed a significant increase in the IL-17 levels compared to the control group. This upsurge in proinflammatory markers highlights a comprehensive inflammatory condition, potentially exacerbating both the metabolic and reproductive complications of the syndrome (Rudnicka et al. 2021). Several factors contributed to this inflammatory state. For instance, adipose tissue, particularly in individuals with PCOS, can release pro-inflammatory cytokines, thereby promoting an inflammatory environment (Vasyukova et al. 2023).

IL-4 is an anti-inflammatory cytokine involved in the Th2 immune response. In PCOS, the levels of IL-4 and IL-10 were reduced compared to the control group. Among these, IL-1RA, IL-10, and IL-4 are the most studied in PCOS (Popovic et al. 2019). The decrease in anti-inflammatory cytokines in PCOS may be a compensatory response to an increase in pro-inflammatory cytokines, as previously reported (Vasyukova et al. 2023). In a contrary study, elevated IL-10 levels in PCOS cases were reported by Talaat et al. (2016). A small sample size limited this work. Furthermore, individuals with PCOS have higher concentrations of proinflammatory interleukins, particularly IL-1α and IL-1β (Palomo et al. 2015).

Understanding the influence of inflammation and immune disturbances in PCOS can inform about the treatment modalities. Research has focused on the use of herbal treatments for PCOS (Chitra and Dhivya 2017). The potential of immunomodulatory therapy is also a promising area for research and therapeutic exploration (Ryssdal et al. 2023). Thus, the widespread inflammatory and altered immune reactions are central to the onset and progression of PCOS (Montanino Oliva et al. 2018). The results presented here highlight the impact of various interventions such as probiotics, myo-ins, *U. dioica*, and *W. somnifera* on inflammatory and anti-inflammatory cytokines in a PCOS-induced immature rat model.

Both the probiotics and myo-ins pre-treatments reduced the TNF-α levels compared to the PCOS group, with the probiotics demonstrating a more pronounced effect. The probiotics showed the most pronounced effects in restoring anti-inflammatory cytokines (IL-10) and reducing pro-inflammatory markers such as TNF-α and IL-17, highlighting their potential as a therapeutic intervention in PCOS. Similarly, myo-ins demonstrated significant immunomodulatory effects, particularly in reducing TNF-α, consistent with its known benefits in PCOS management. Probiotics and other microbiota-modulating therapies may be useful in the treatment of PCOS. In recent decades, a growing body of research has been conducted on the gut microbiota and its advantageous role in the host’s immunological, nutritional, and metabolic processes (Ejtahed et al. 2020). Recent research suggests a link between the gut microbiota and PCOS aetiology. Thus, probiotic-modulating therapies may be useful in the treatment of PCOS (Wang et al. 2021). The gut microbiota prevents the formation of harmful microbes and reduces inflammation and gut leakage (Ejtahed et al. 2019). By altering the gut microbiota, increasing the amounts of *Bifidobacteria* and *Lactobacilli*, re-establishing the microbiota balance, decreasing intestinal permeability, and lowering the translocation of lipopolysaccharides from the intestine to the blood circulation, probiotics have been approved to alleviate the symptoms of PCOS (Xue et al. 2019).

Agents with anti-inflammatory properties like myo-ins have demonstrated efficacy in mitigating PCOS manifestations, likely by addressing the underlying inflammatory condition (Xia et al. 2023). Inositols are considered insulin sensitisers because they alter the components of the insulin signalling pathways (Dinicola et al. 2021). They have a favourable impact on the menstrual cycle regularity, carbohydrate metabolism, and the clinical and laboratory manifestations of hyperandrogenism (Pundir et al. 2018). However, there is currently insufficient data to support their inclusion in the guidelines as standard therapy (Teede et al. 2018). There is some evidence indicating that patients with PCOS have highly dysregulated inositol metabolism in their follicular cells (Heimark et al. 2014). In contrast, inositol enhances ovarian function and oocyte quality by modulating several hormonal pathways (Bevilacqua and Bizzarri 2016). However, a therapeutic formula with myo-ins proved to be ineffective or even adversely affected clinical-pathological outcomes (Bevilacqua et al. 2019).

The *U. dioica* group exhibited a substantial reduction in IL-17, indicating its potent anti-inflammatory properties. Similarly, *W. somnifera* also reduced the TNF-α levels, supporting its use as an adaptogen with immunomodulatory effects. A nettle extract may be a beneficial supplement to improve the immunological and histological abnormalities associated with PCOS. Together, these findings suggest that further investigation into combining these interventions may offer a synergistic approach to managing PCOS-associated inflammation and immune dysregulation. Future studies should explore the mechanistic pathways through which these treatments exert their effects and assess their long-term efficacy and safety in PCOS animals.

The pathogenesis of PCOS involves not only cytokine disturbance but also heightened OS, which leads to various outcomes (Shorakae et al. 2018). The data provide insights into the impacts of various interventions on the antioxidant activities in the serum and liver extracts of a PCOS rat model. OS refers to an imbalance between the production of reactive oxygen species (ROS) and the body’s antioxidant defences (Adeyanju et al. 2020). OS refers to a state in which there is a discrepancy between the production of ROS and the capacity of a biological system to neutralise these reactive entities or repair the resulting damage (Adwas et al. 2019). In normal physiological states, ROS emerge as secondary products of mitochondrial electron transport processes and are instrumental in cellular signalling and maintaining equilibrium (Dutta et al. 2020). Antioxidants like CAT, GSH-Px, and SOD are key enzymatic defence that mitigates the oxidative damage in cells and tissues (Lu et al. 2018). A better understanding of the functions of these enzymes may clarify the pathomechanisms of various reproductive illnesses and potentially pave the way for novel therapeutic approaches.

PCOS is known to disrupt the metabolic and hormonal balance, leading to increased oxidative stress biomarkers and impaired antioxidant defences, as evidenced by the significantly lower serum and liver extract levels of CAT, SOD, and GSH-Px in the PCOS group compared to the control group. However, as the relationship between the antioxidant status and PCOS becomes more apparent, it is still necessary to explore the exact mechanisms and determine the optimal course of action. In a rat model of PCOS, antioxidant and anti-inflammatory polyphenol lowers the levels of OS, lipid peroxidation, inflammatory cytokines, and DNA oxidative damage in ovarian tissue (Mazloom et al. 2019). Moreover, the chronic inflammatory response might be attributed to the detrimental outcomes of OS (Mohammadi 2019). Recognising the significance of OS in PCOS is crucial, both for understanding the foundational pathophysiological aspects of the condition and for the creation of therapeutic strategies addressing this oxidative discrepancy (Sengupta et al. 2023). Some upcoming research may introduce antioxidant-focused therapies or lifestyle modifications aimed at reducing the oxidative stress biomarkers in individuals with PCOS (Sengupta et al. 2023). Dutta et al. (2024) emphasise the crucial link between OS and female reproduction and offer suitable treatment options.

All four treatments used in the current study showed various improvements in the antioxidant enzyme activities in the serum and liver extracts compared to the untreated PCOS group. Myo-ins both showed significant benefits in enhancing the CAT activity, supporting their use as therapeutic agents in managing PCOS. Discoveries in recent decades have opened up new possibilities in the clinical management of PCOS by increasing the tissue availability of myo-inositol and reversing abnormalities observed in inositol-based pathways (Facchinetti et al. 2015). Herbal extracts, particularly *U. dioica*, demonstrated strong antioxidant (SOD) activities, suggesting their potential as a natural treatment for oxidative stress and metabolic disturbances in PCOS. *W. somnifera* also showed positive effects, though to a lesser extent than *U. dioica*. Future therapeutic approaches that focus on mitigating OS may offer potential solutions for the reproductive complications of this condition (Uckan et al. 2022). Sengupta et al. (2024) collected the current knowledge on the interactions between PCOS and OS, highlighting potential treatment options and the importance of more research to clarify this complex relationship. Since ancient times, herbal plants have been a major source of medicinal treatments. Remarkably, despite changes in the pharmaceutical industry, the use of herbal remedies is growing daily in developing nations (Krishna et al. 2023). Probiotics showed some improvements across all the parameters, although their effects were slightly less potent than those of the other interventions. Nonetheless, probiotics offer a promising means to alleviate oxidative stress in PCOS. These findings suggest that combining these treatments could provide a comprehensive approach to managing both the oxidative stress biomarkers and metabolic aspects of PCOS.

The histological examination of the ovaries of the TP-treated rats showed the presence of a follicular cyst and significant damage to some mature follicles and their associated cells, consistent with previously established models (Chaudhari and Nampoothiri 2017). The administration of TP appears to have a suppressive effect on the lymphoid tissue within the spleen. This lymphoid depletion might be due to changes in the immune function. The histological examination indicated that the addition of a probiotic or myo-ins to the TP treatment regimen resulted in a significant improvement in ovarian health. The follicular cysts were resolved, and normal follicular development and corpus luteum formation were observed. The lymphoid follicles within the white pulp of the spleen showed a mild reduction in lymphoid cells in rats treated with TP along with probiotics. This combination appears to have a less severe effect on the lymphoid tissue compared to the group treated with TP alone. Probiotics and myo-ins are beneficial in modulating immune responses.

The histological examination has shown that in the ovaries of rats treated with TP and *U. dioica* extracts, still medium-sized follicular cysts were present, with a normal and intact spleen, indicating that the treatment did not fully address the condition. Studies suggest that nettle extracts reduce the morphological and histological changes associated with PCOS (Bandariyan et al. 2021). Since the nettle has anti-inflammatory and antioxidant properties, we anticipated that using both extracts together would alleviate the negative consequences of PCOS in rats. The majority of these biological processes are thought to be supported by *U. dioica*’s antioxidant action (Lee and Lee 2021). For instance, TNF-α plays a significant role in controlling the ovary’s typical activity during the follicular development stage. However, the formation of cystic follicles could result from an increase in this factor caused by causing apoptosis in the granulosa cells of ovarian follicles (Wang and Zhu 2012). Nettle extract successfully reduced polycystic ovarian morphological and histological alterations as well as the consequences of metabolic syndrome-related sex hormone alterations in a rat model of PCOS (Zare et al. 2015). The combined treatment of TP and *W. somnifera* extracts appears to be highly effective in treating ovarian cysts. *W. somnifera* with its antioxidant resulted in a reasonable level of protection against the TP effect through regularisation of the oestrous cycle (Soujanya et al. 2022).

In conclusion, the use of these four materials as monotherapy in this study led to various changes; therefore, further investigation is recommended to explore combinations of duos or trios of these treatments managing both the oxidative stress biomarkers and metabolic aspects of PCOS. Further research should focus on elucidating the molecular mechanisms underlying these effects and exploring the long-term efficacy of these interventions in clinical settings.
